# Spontaneously Resolving Joint Inflammation Is Characterised by Metabolic Agility of Fibroblast-Like Synoviocytes

**DOI:** 10.3389/fimmu.2021.725641

**Published:** 2021-08-26

**Authors:** Jane Falconer, Valentina Pucino, Sally A. Clayton, Jennifer L. Marshall, Sabrina Raizada, Holly Adams, Andrew Philp, Andrew R. Clark, Andrew Filer, Karim Raza, Stephen P. Young, Christopher D. Buckley

**Affiliations:** ^1^Rheumatology Research Group, Institute for Inflammation and Ageing, College of Medical and Dental Sciences, University of Birmingham, Queen Elizabeth Hospital, Birmingham, United Kingdom; ^2^School of Medicine, Institute of Health Sciences and Wellbeing, University of Sunderland, Sunderland, United Kingdom; ^3^Institute of Metabolism and Systems Research, College of Medical and Dental Sciences, University of Birmingham, Birmingham, United Kingdom; ^4^Healthy Ageing Theme, Garvan Institute of Medical Research, Darlinghurst, NSW, Australia; ^5^St Vincent’s Clinical School, UNSW Medicine, UNSW Sydney, Sydney, NSW, Australia; ^6^Department of Rheumatology, Sandwell and West Birmingham NHS Trust, Birmingham, United Kingdom; ^7^Kennedy Institute of Rheumatology, University of Oxford, Oxford, United Kingdom

**Keywords:** fibroblasts, arthritis, inflammation, metabolism, mitochondria

## Abstract

Fibroblast-like synoviocytes (FLS) play an important role in maintaining joint homeostasis and orchestrating local inflammatory processes. When activated during injury or inflammation, FLS undergo transiently increased bioenergetic and biosynthetic demand. We aimed to identify metabolic changes which occur early in inflammatory disease pathogenesis which might support sustained cellular activation in persistent inflammation. We took primary human FLS from synovial biopsies of patients with very early rheumatoid arthritis (veRA) or resolving synovitis, and compared them with uninflamed control samples from the synovium of people without arthritis. Metabotypes were compared using NMR spectroscopy-based metabolomics and correlated with serum C-reactive protein levels. We measured glycolysis and oxidative phosphorylation by Seahorse analysis and assessed mitochondrial morphology by immunofluorescence. We demonstrate differences in FLS metabolism measurable after *ex vivo* culture, suggesting that disease-associated metabolic changes are long-lasting. We term this phenomenon ‘metabolic memory’. We identify changes in cell metabolism after acute TNFα stimulation across disease groups. When compared to FLS from patients with early rheumatoid arthritis, FLS from patients with resolving synovitis have significantly elevated mitochondrial respiratory capacity in the resting state, and less fragmented mitochondrial morphology after TNFα treatment. Our findings indicate the potential to restore cell metabotypes by modulating mitochondrial function at sites of inflammation, with implications for treatment of RA and related inflammatory conditions in which fibroblasts play a role.

## Introduction

In health, quiescent fibroblast-like synovial cells (FLS) are anabolic, producing abundant collagen and hyaluronic acid and maintaining homeostasis and turnover of synovium and cartilage. However, in disease, stromal and myeloid populations expand and leukocytes are recruited to the synovial tissue contributing to an increasingly inflammatory and hypoxic environment (1, 2). In the transformation to a chronically activated and pathogenic phenotype, FLS respond and contribute to these environmental cues, forming an invasive pannus tissue and producing degradative enzymes which damage cartilage and bone. These cellular behaviours and microenvironmental features closely resemble those seen in malignancy, where they are associated with therapeutically targetable changes in nutrient and oxygen availability, metabolic demand and metabolic phenotype of cells (3, 4).

In rheumatoid arthritis (RA) patients, metabolic deviation is observed globally as an increase in basal metabolic rate (5) and symptoms of cachexia. This phenotype is thought to be orchestrated by inflammatory cytokines such as TNFα, IL-1β, IL-6, LIF, and IFNγ which are elevated in chronic inflammatory diseases (6, 7). In addition multiple cellular metabolic dysfunctions have been identified (8–10). We and others have shown that metabolic fingerprints measurable in body fluids (serum, urine and synovial fluid) correlate with hallmarks of diseases including CRP and TNFα levels, and can predict drug responses (11–15). Metabolomic studies such as these have the power to identify novel biomarkers and drug targets but have less frequently been used to investigate pathological changes in metabolism at a cellular level or the contribution of individual cell types to the overall metabolic phenotype of RA (16).

Aerobic glycolysis, a characteristic response of many cell types to pro-inflammatory stimuli, is defined by the metabolic fate of pyruvate, a product of glucose catabolism. Rather than being converted to acetyl-CoA and used to fuel mitochondrial respiration, in aerobic glycolysis pyruvate is instead reduced to lactate and exported from the cell. In RA, FLS are reported to undergo several metabolic alterations, including an increase in aerobic glycolysis (17–19); impaired mitochondrial respiration and altered mitochondrial dynamics (20, 21); as well as altered lipid metabolism (22). However, to date these studies have used mouse models of disease or tissue from very late stage RA and osteoarthritis and have not explored the mechanisms responsible for the persistence rather than resolution of inflammatory disease. It is well-established that early intervention in the course of disease progression is important for preventing joint damage and dysfunction in RA (23). Therefore knowledge of the metabolic state in early disease is critical for understanding both disease mechanisms and possible treatment options that target metabolic components.

In this study, we describe the metabolic fingerprint of human resting and primed FLS from uninflamed and inflamed joints and elucidate a metabolic phenotype of FLS which distinguishes acute, self-limiting synovitis (resolving arthritis) from very early, persistent RA (veRA).

## Methods 

### Patients

The study was conducted in compliance with the Declaration of Helsinki. Ethical approval was obtained from the local ethics committee and all subjects provided written, informed consent. Patients with early arthritis were seen in the BEACON cohort, details of which have been reported previously (24). Unselected, consecutive DMARD- and glucocorticoid-naïve patients with at least one clinically swollen joint within 12 weeks of the onset of any inflammatory symptoms were recruited and followed for 18 months to determine diagnostic outcome. Age and sex matched patients were classified as having very early, persistent RA (veRA) according to the 2010 ACR criteria (25). As previously described, patients with resolving arthritis were diagnosed if there was no evidence of joint related soft-tissue swelling on final examination and where no DMARD or steroid treatment was administered in the preceding 3 months (26). Consenting patients with appropriate joints underwent ultrasound-guided synovial biopsy using needle or portal and forceps techniques (27, 28). NSAID usage and ultrasound-derived inflammation were comparable between disease groups. The uninflamed control group comprised patients with no evidence of degenerative or inflammatory disease, macroscopic or microscopic joint pathology, who underwent exploratory conventional arthroscopy for knee pain. All patients underwent detailed clinical and laboratory evaluations to rule out any concomitant inflammatory, metabolic, and neoplastic disorders. FLS were maintained in culture *ex vivo* and all lines were at the same passage number when experiments were performed.

### FLS Culture

FLS were grown out of tissue and maintained at 70-80% confluence in media containing 10% fetal calf serum, MEM Non-essential amino acids (0.87x), sodium orthopyruvate (0.87 mM), glutamine (1.75mM), penicillin (87U/ml) and streptomycin (87ug/ml). After 3 passages, cultures were >99.5% phenotypically homogeneous. Conditioned culture medium and cells were harvested for bioenergetic analysis, immunofluorescence staining or NMR spectroscopy at passage 6 and 5-6 cell lines from each disease group were utilized in each individual experiment.

### Metabolite Preparation

For preparation of metabolites from conditioned culture medium, supernatants were collected, centrifuged at 13000g for 5 minutes and filtered to exclude species >3KDa (29). Flow through was mixed in a 1:1 ratio with an aqueous NMR buffer with final concentrations of 10% D_2_O, 150mM NaCl, 1mM trimethylsilyl 2,2,3,3-tetradeuteropropionic acid (TMSP) and 20mM sodium phosphate (pH7). Intracellular metabolites were extracted using cold methanol/chloroform (30). The aqueous fraction was dried overnight and resuspended in NMR buffer as described above.

### Metabolomic Analysis

One dimensional (1D) ^1^H spectra were acquired at 300K using a standard spin-echo pulse sequence with water suppression and using excitation sculpting, on a Bruker DRX 600MHz NMR spectrometer equipped with a cryoprobe. Samples were processed and data calibrated with respect to the TMSP signal. Spectra were read into ProMetab, custom written software in Matlab (version 7, The Mathworks, Natick, MA) and truncated to a chemical shift range of 0.8-10.0 ppm (31). The water peak was removed, spectra were corrected for baseline offset and normalised to a total spectral area of unity, and a generalised log transformation was applied (31, 32). Spectra were then read into Chenomx NMR suite (Chenomx, professional version 4.0) and an inbuilt peak database was used alongside published data to identify and quantify associated metabolites (33). Pathway analysis was carried out using Metaboanalyst 3.0 software.

Partial least‐squares regression analysis (PLS‐R) identifies metabolites which predict a continuous variable, and was used to investigate the relationship between the metabolic fingerprint and inflammatory burden measured as C‐reactive protein (CRP). This analysis yielded an R^2^ value as a measure of the cross‐validated goodness‐of‐fit of the linear regression. Permutation testing was used to assess the statistical significance of the relationship when compared to a slope of zero.

### Cellular Bioenergetics

FLS were seeded in 24 well flux plates (Seahorse) at 2x10^4^/well and allowed to adhere for 24 hours in the presence or absence of 1ng/ml TNFα. Prior to assay, cells were equilibrated for 1 h in a non-CO_2_ incubator at 37°C. Cellular bioenergetics were analyzed within the University of Birmingham Mitochondrial Profiling Facility using a Seahorse XFe24 extracellular flux analyzer according to the manufacturer’s instructions. Both oxygen consumption rate (OCR, as a measure of oxidative phosphorylation) and extracellular acidification rate (ECAR, as a measure of aerobic glycolysis), were assessed. ECAR was measured in XF media in basal condition and in response to 10 mmol/L glucose (basal glycolysis), 2 μmol/L oligomycin (glycolytic capacity) and 50 mmol/L 2-DG. OCR was measured in XF media (non-buffered DMEM medium, containing 10 mmol/L glucose, 2 mmol/L l-glutamine, and 1 mmol/L sodium pyruvate), under basal conditions (basal respiration) and in response to 2 μmol/L oligomycin (ATP-linked respiration), 5 μmol/L of carbonylcyanide-4-(trifluoromethoxy)-phenylhydrazone (FCCP) (maximal respiration) and 3 μmol/L antimycin and rotenone (Sigma Aldrich). Three technical replicates were carried out for each condition and a total of 17 measurements of 4 minutes duration were made. Calculations of glycolysis and respiration were established from area under the curve.

### Immunofluorescence and Mitochondrial Analysis

Cells were adhered to chamber slides at 2x10^3^/well and cultured with or without 1ng/ml TNFα for 24 hours. Slides were fixed in acetone, air-dried and stored at -20°C. Slides were rehydrated in PBS, blocked in 10% normal goat serum and incubated with mouse anti-TOMM20 (4F3, Abcam, UK) prior to goat anti-mouse IgG1 (Southern Biotech, Birmingham, USA). The FITC signal was amplified with anti-FITC Alexa Fluor 488 (Life Technologies) and nuclei were stained with Hoechst 33258. Slides were mounted in Prolong Diamond (Life Technologies) before imaging. Images were captured on the Leica DM6000 using the proprietary software and processed using Fiji (34) in a method adapted from (35). In brief, the image in the TOMM-20 channel was sharpened, thresholded, converted to a mask and then skeletonized prior to running the binary connectivity plug-in as described (36, 37). For visualisation the Glasbey lookup table was used and numbers of each pixel connection type were exported. Nuclei were counted as an assessment of cell number in each field of view and these data were combined.

### Statistical Analysis

Data are presented as mean ± SEM. Analysis of variance (ANOVA) was used for multiple comparisons, paired Student’s t tests for comparison of resting and stimulated cells. Results were considered significant where p<0.05.

## Results

### Steady State Metabolomic Analysis in FLS From Healthy and Inflamed Joints

Patients presenting with synovial inflammation underwent synovial biopsy at presentation and were subsequently classified as resolving arthritis or very early RA (veRA) (24), and were compared with uninflamed synovial samples obtained from exploratory arthroscopy as detailed in methods. Clinical characteristics of subjects are shown in **Table 1**.

**Table 1:** Demographics and clinical characteristics of participants at the time of synovial biopsy.

	Uninflamed controls (n = 11)	Resolving arthritis (n = 12)	Very early RA (n = 11)
Age (years); median (IQR)	41 (38-44)	40.5 (32.25-52.5)	61 (48-70)
Female; number (%)	5 (45.5)	5 (41.7)	6 (54.5)
Symptom duration (weeks);median (IQR)	–	5 (3.25-6.75)	4 (3.5-7.5)
NSAID; number (%)	0 (0)	9 (75.0)	8 (72.7)
CRP (mg/ml); median (IQR)	–	8.5 (1.5-13)	25 (10-32)
RF positive; number (%)	–	0 (0)	5 (45.5)
Anti CCP antibody positive; number (%)	–	0 (0)	7 (63.6)
Joint biopsied			
Ankle; n (%)	0 (0)	3 (25)	3(27.3)
Knee; n (%)	11 (100)	9 (75)	5 (45.4)
MCP; n (%)	0 (0)	0 (0)	3 (27.3)
Ultrasound greyscale hypertrophy score (1-3); median (IQR)	–	2 (1-2)	3 (1.75-3)
Ultrasound Power Doppler hypertrophy score (1-3); median (IQR)	–	1(1-2)	1.5 (0.75-2)

RA, rheumatoid arthritis; IQR, interquartile range; NSAID, non-steroidal anti-inflammatory drugs; CRP, C-reactive protein; RF, rheumatoid factor; CCP, cyclic citrullinated peptide; MCP, metacarpopharangeal. Of these patients, cells from 5-6 individuals from each disease group were used in each experiment.

**Figure 1:**
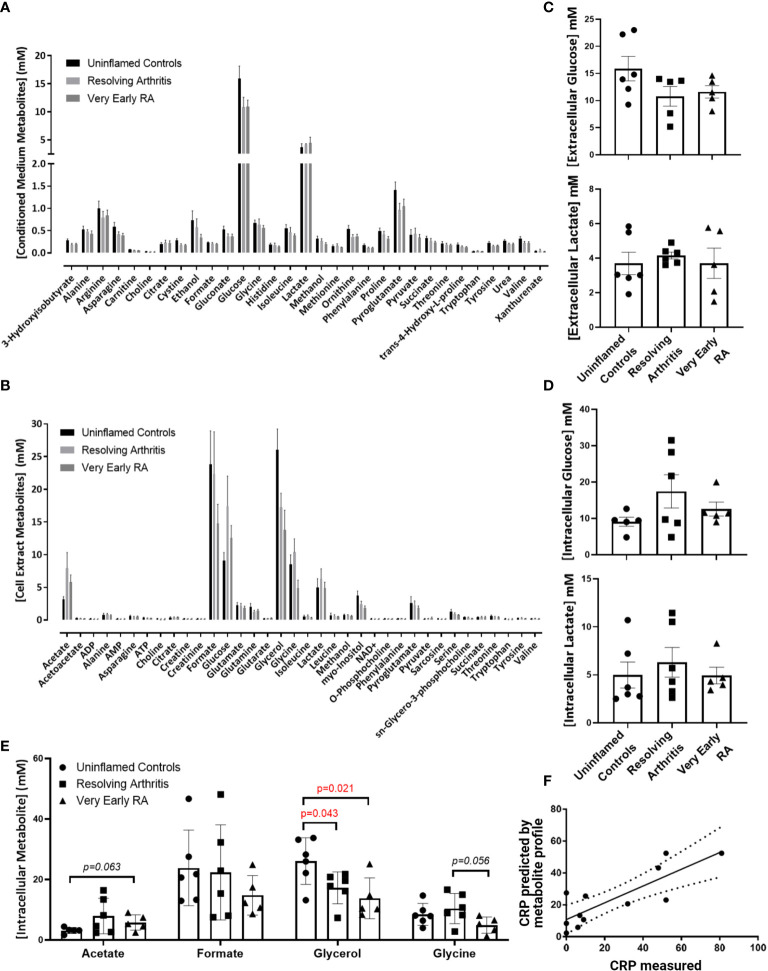
Metabolomic fingerprinting in FLS from uninflamed and inflamed synovium. FLS were cultured from synovial biopsies of patients with no inflammation (n=6), resolving arthritis (n=6) and very early rheumatoid arthritis (RA) (n=5) and metabolites were quantified by 1D nuclear magnetic resonance spectroscopy. **(A)** All quantified metabolites measured in conditioned culture medium, **(B)** All quantified metabolites measured in cell extracts, **(C)** glucose and lactate measured in conditioned culture medium, **(D)** glucose and lactate measured in cell extracts and **(E)** other metabolites for which differences were observed between disease groups, **(F)** strong correlation between measured C-reactive protein (CRP) levels and CRP levels predicted by the FLS metabolic profile in uninflamed controls and arthritis patients. The predicted values were calculated from the concentrations of metabolites identified using partial least squares regression analysis (R^2^ = 0.6801). Statistical significance was determined by one-way ANOVA test.

To assess whether alterations in the bioenergetic responses of FLS drive the pathological transition to chronicity in RA we started our analyisis by assessing the metabolomic profile in FLS derived from uninflamed, resolving synovitis and RA patients. 1D NMR spectroscopy was carried out on 6 FLS cell lines derived from uninflamed patient synovium, 6 FLS cell lines from resolving arthritis patients and 5 FLS cell lines from veRA patients. We identified metabolic signatures from these cells and identified 31 metabolites present in all 17 conditioned media (**Figure 1A**) and a further 36 present in all 17 cell extracts (**Figure 1B**). A summary of pathways which these data implicate as important in FLS metabolism is shown in **Supplementary Figure 1**. Glycolysis is an important metabolic pathway that utilises glucose for biosynthesis and ATP generation. Lactate, which is the end product of glycolysis, and glucose were most highly represented in culture supernatants of all patient groups (**Figures 1A, C**). Glucose and lactate levels in supernatants and cell extracts did not vary between disease groups (**Figures 1C, D**).

Glycerol was significantly reduced in cell extracts of resolving and veRA synovitis when compared to uninflamed controls (**Figure 1E**). Glycerol is a major link between sugar and fatty acid metabolism (38) by reducing dihydroxyacetone phosphate (DHAP, a key triose in glucose metabolism and energy generation) into glycerol-3-phosphate, which is suggestive of a potential substrate to feed lipid synthesis.

Acetate, which is also involved in lipid metabolism, showed higher trend in veRA synovitis relative to controls (p=0.06) while the amino acid glycine was at lower concentrations in FLS extracts from veRA than resolving arthritis, however this did not reach statistical significance (p=0.056, **Figure 1E**).

Although we did not identify striking differences in individual metabolites between groups, we went on to investigate whether the metabolites measured by NMR spectroscopy were linked to the inflammatory status of individuals at the time of biopsy using PLS-R analysis (**Figure 1F**). Indeed, a metabolomic profile was identified which can predict levels of the inflammatory marker C-reactive protein (CRP) in patient sera (R^2^ = 0.6801, p=0.001).

### Differential Metabolic Adaptation in FLS From Resolving *Versus* Persistent Inflamed Synovium

**Figure 2:**
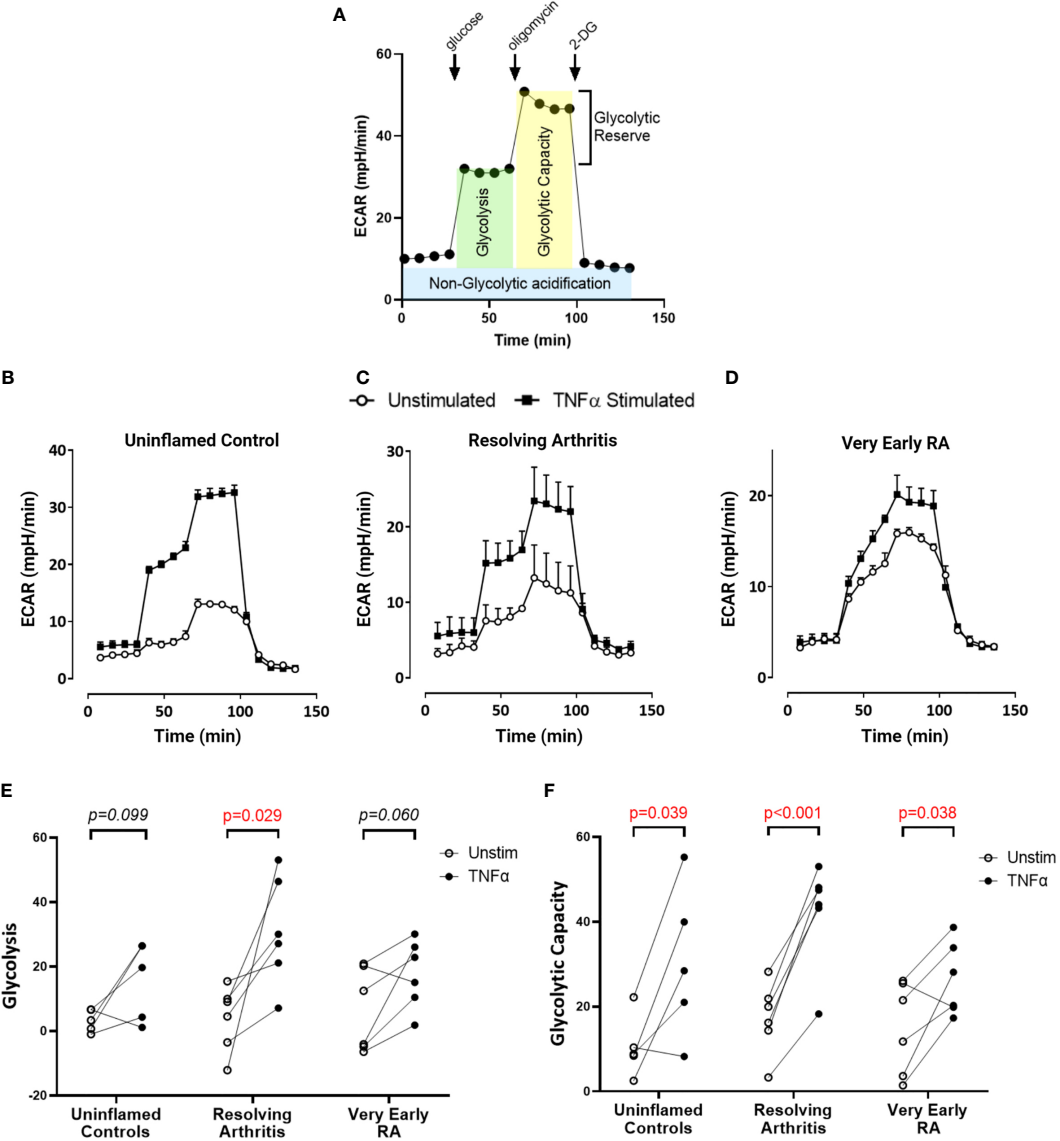
FLS from healthy and inflamed synovium show increased glycolytic capacity in response to TNFα. FLS were cultured from synovial biopsies of patients with no inflammation (n=5), resolving arthritis (n=6) and very early rheumatoid arthritis (RA) (n=6). Extracellular acidification rate (ECAR) was measured in real time in the presence of glucose, oligomycin and 2-deoxyglucose (2DG) **(A)**. Example traces are shown for unstimulated FLS and FLS stimulated for 24 hours with tumour necrosis factor α (TNFα); **(B)** uninflamed, **(C)** resolving arthritis and **(D)** very early RA. **(E)** Glycolysis (after the addition of glucose) and **(F)** glycolytic capacity (difference of oligomycin rate and 2DG rate) were calculated as shown in **(A)**. Statistical significance between treatments was determined by paired student’s t test. No statistical significance was found between patient groups.

**Figure 3:**
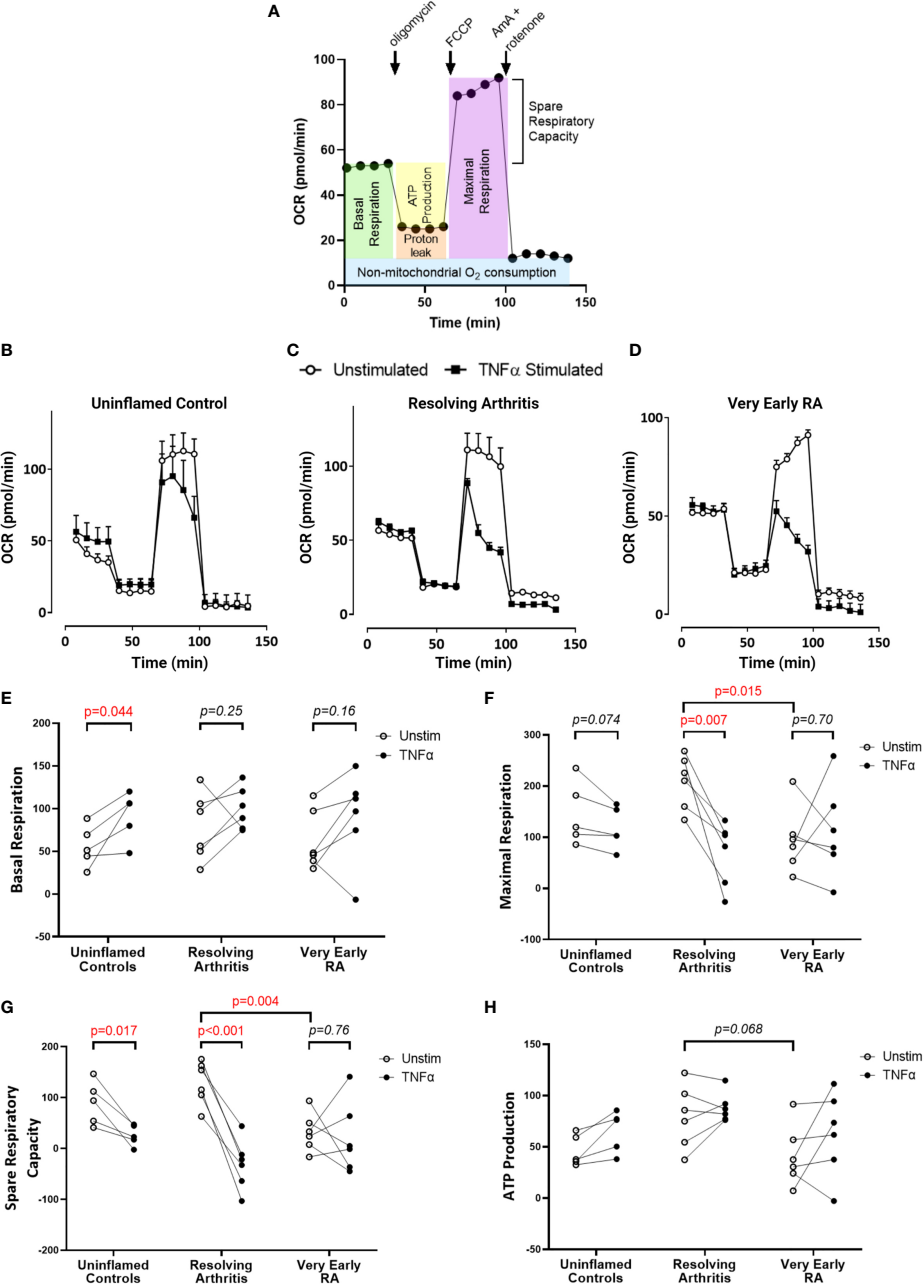
FLS from patients with very early RA have reduced respiratory capacity compared with those from patients with resolving arthritis. FLS were cultured from synovial biopsies of patients with no inflammation (n=5), resolving arthritis (n=6) and very early rheumatoid arthritis (RA) (n=6). Oxygen consumption rate (OCR) was measured in real time in the presence of oligomycin, carbonyl cyanide-p-trifluoromethoxyphenylhydrazone (FCCP) and antimycin A (AmA) and rotenone in combination **(A)**. Example traces are shown for unstimulated FLS and FLS stimulated for 24 hours with tumour necrosis factor α (TNFα); **(B)** uninflamed, **(C)** resolving arthritis and **(D)** very early RA. Basal respiration rate [**(E)**, before addition of oligomycin], maximal respiration rate [**(F)**, difference of FCCP rate and AmA + rotenone rate], spare respiratory capacity [**(G)**, difference between the rate of basal respiration and the maximum, FCCP-stimulated rate of respiration], and ATP-linked respiration [**(H)**, ATP production, difference of basal rate and oligomycin rate] were calculated as shown in **(A)**. Statistical significance between treatments was determined by paired student’s t test. Statistical significance between groups was determined by two-way ANOVA.

To directly investigate the balance of glycolytic and mitochondrial energy generation in resolving arthritis and veRA, we used the Seahorse XF Analyzer and metabolic inhibitors or potentiators to determine the maximal capacity of FLS to utilize these bioenergetic pathways under stress. The parameters calculated from the Seahorse glyco- and mito-stress tests are detailed in **Figures 2A** and **3A**. Seahorse analysis was carried out on 5 FLS cell lines derived from uninflamed synovium, 6 FLS cell lines from resolving arthritis patients and 6 FLS cell lines from veRA patients. We assessed both resting and TNFα stimulated FLS. Example traces measuring extracellular acidification rate (ECAR) as a measure of aerobic glycolysis and oxygen consumption rate (OCR) as a measure of mitochondrial respiration in each patient group are shown in **Figures 2B–D** and **3B–D** respectively.

Basal glycolysis was comparable in all disease groups (**Figure 2E**), consistent with the metabolomic data showing no change in glucose uptake and lactate production in the resting state (**Figures 1C, D**). Interestingly only resolving arthritis FLS showed a significant upregulation of glycolysis in response to TNFα (**Figure 2E**). Glycolytic capacity is determined using the mitochondrial ATP synthase inhibitor oligomycin (**Figure 2A**), and demonstrates the cell’s maximal possible rate of glycolysis under the given conditions. Glycolytic capacity was significantly enhanced by TNFα treatment in all groups (**Figure 2F**)

Respiratory parameters were determined using oligomycin to inhibit ATP synthase; FCCP to uncouple oxygen consumption from ATP synthase; and rotenone plus antimycin A to poison mitochondrial respiration (**Figure 3A**). Basal respiration in resting FLS did not differ between patient groups, and was significantly increased by TNFα only in healthy control FLS (**Figure 3E**). Maximal respiration was significantly higher in resolving arthritis than veRA FLS, and was significantly reduced by TNFα treatment only in resolving arthritis FLS (**Figure 3F**). Spare respiratory capacity (the difference between maximal and basal respiration rates) was significantly higher in resolving arthritis than veRA FLS, and was significantly reduced by TNFα treatment in healthy control and resolving synovitis FLS, but not in veRA FLS (**Figure 3G**). Differences in respiration linked to ATP synthesis were not significant (**Figure 3H**). Thus, FLS from patients with resolving synovitis or early arthritis differed in certain basal metabolic parameters even after several passages, providing further evidence of sustained metabolic memory. The capacity of metabolic activities to respond to an inflammatory challenge was characteristic of FLS derived from patients with resolving synovitis, but lost in patients who would later be diagnosed with RA. These results suggest that mitochondrial dysfunction in FLS is an early event in RA disease, which may be linked to the development of persistent synovitis. Whether this reduced capacity is driven by specific inflammatory signals within the RA synovial environment remains to be determined.

### Resolving FLS Display a Distinct Mitochondrial Morphology in Response to TNFα

**Figure 4:**
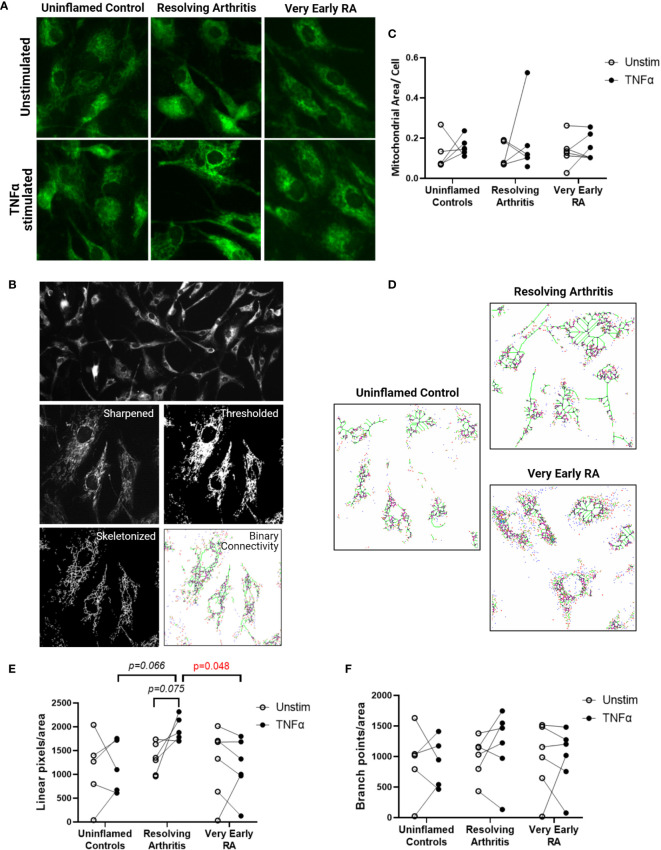
FLS from resolving arthritis synovium display a distinct mitochondrial morphology in comparison to healthy and RA FLS in response to stimulation. FLS from patients with no inflammation (n=5), resolving arthritis (n=6) and very early rheumatoid arthritis (RA) (n=6) were labelled with TOMM-20 and visualised by fluorescence microscopy **(A)**. **(B)** Representative image showing how TOMM-20 channel was sharpened, thresholded, converted to a mask and then skeletonized prior to running the binary connectivity plug-in. **(C)** Mitochondrial area per cell was quantified in unstimulated cells and cells stimulated with TNFα for 24 hours. **(D)** Representative images of mitochondrial connectivity in TNFα-stimulated FLS from different disease groups. Filamentous/linear (green) or fragmented/punctate (purple) mitochondria are represented (39). Quantification of linear pixels **(E)** and branch point pixels **(F)** per cell normalised to mitochondrial area from skeletonized images of unstimulated cells and cells stimulated with TNFα for 24 hours. Statistical significance between groups was determined by one-way ANOVA test.

We next investigated whether the metabolic differences observed above were associated with changes in mitochondrial morphology and dynamics. Immunofluorescence imaging was carried out on 5 FLS cell lines derived from uninflamed synovium, 6 FLS cell lines from resolving arthritis patients and 6 FLS cell lines from veRA patients. FLS mitochondria were visualized by staining for TOMM20 (**Figure 4A**), and mitochondrial area, linearity and branching were quantified (**Figure 4B**). Total mitochondrial area per cell varied between individuals and was comparable between disease outcomes and unchanged after stimulation of cells with TNFα for 24 hours (**Figure 4C**). Linearity and branching also showed great variability between individuals (**Figures 4D–F**). However, FLS from patients with resolving arthritis showed increased mitochondrial linearity following TNFα treatment in comparison to the other groups (**Figure 4E**). Consistent with the results of the bioenergetic analysis, measurement of mitochondrial linearity also revealed that the FLS from resolving arthritis patients demonstrated the greatest degree of plasticity in response to TNFα stimulation (**Figure 4E**). We therefore conclude that the capacity to spontaneously resolve synovial inflammation is associated with elevated metabolic agility of fibroblast-like synoviocytes.

## Discussion

Altered metabolism and dysregulated metabolic pathways are important determinants of inflammatory and destructive processes in chronic inflammatory and autoimmune diseases, including rheumatoid arthritis (RA) (1, 8–10, 40). In this study we explored the hypothesis that alterations in the bioenergetic responses of fibroblast-like synoviocytes (FLS) may be important in orchestrating the pathological transition to chronicity in RA. As the FLS were taken from patients who had a symptom duration of less than 3 months, our analysis gives unique insights into the phenotypic changes that fibroblasts undergo during the initiation of, or adaptation to inflammation. We found that FLS from patients with synovitis that went on to resolve (resolving arthritis) showed greater mitochondrial respiratory capacity than those from patients with very early, persistent RA (veRA), and presented with greater mitochondrial linearity upon morphological analysis. Resolving arthritis FLS also consistently demonstrated greater metabolic agility in response to inflammatory stimulation with TNFα *ex vivo*. These results suggest that the ability of FLS to metabolically adapt to their environment and to carry out mitochondrial respiration may contribute to their capacity to resolve inflammation, and that potentiating mitochondrial function could be a novel strategy to promote resolution of arthritis.

We identified a signature of 36 metabolites common to FLS regardless of disease status, highlighting pathways fundamental to the physiological function of the FLS. We observed that in particular glucose and lactate levels were not differerent between groups. These findings suggest that FLS undertake glycolytic metabolism even in the absence of inflammation. This is an important consideration given the recent interest in targeting glycolytic pathways for the treatment of autoimmune diseases including RA, however we acknowledge the potential differences between *in vitro* and *in vivo* metabolic commitments.

It is also likely that a similar metabolic profile supports the shared functions of fibroblasts at sites other than the joint, as demonstrated by findings in foreskin-derived cells (41). These cells were heavily reliant upon glucose metabolism and utilised the pentose phosphate pathway to maintain a biosynthetic phenotype even in quiescence (41). Although studies report upregulation of glucose transporters in inflammation (17, 42), we did not find differences in terms of glucose uptake and lactate production from culture medium in FLS from both resolving arthritis and veRA patients *in vitro*. Our NMR analysis in early pathogenesis is in line with a mass spectrometry based study of FLS which identified disturbance in metabolites associated with amino acid, sugar and lipid metabolism compared to osteoarthritis, but which did not identify a switch to glycolysis even in end stage RA FLS (43).

Glycerol depletion in cells from both resolving arthritis and veRA patients suggests there is also a role for lipid metabolism in the steady state and emphasises the importance of upregulation of this pathway during the inflammatory response as well as in the resolution of inflammation as previously described (44). It would be valuable to perform a lipidomic study in the future to further clarify this mechanism.

Building on these metabolomic findings using Seahorse bioenergetic analysis, we again showed no disease-associated augmentation of glycolysis. This suggests that the early stages of the disease are not characterised by commitment to glycolysis, for example as a result of epigenetic reprogramming, mitochondrial dysfunction or loss. Enhanced glycolysis may be a lasting adaptation to mitochondrial damage or to low oxygen supply (45) as is found in the inflamed joint (2). A permanent switch toward aerobic glycolysis is important to the pathogenic phenotype of tumour cells and cancer-associated fibroblasts (46), and has been described in some but not all studies of late stage RA FLS metabolism (19, 40) however our results show that this does not occur in early stages of arthritis (**Figure 2E**).

Alternatively, an upregulation of glycolysis can be a transient, physiological response to activation stimuli, as is well characterised in innate and adaptive immune cells (47). A glycolytic response in fibroblasts exposed to hypoxia (19) platelet derived growth factor (PDGF), lipopolysaccharide (LPS) (17) as well as complement (48) has been documented. An early study demonstrated upregulation of glycolysis in TNFα stimulated, late stage RA FLS (49).

Due to their metabolic agility, we showed that resolving FLS after re-exposure to TNFα were better equipped to increase glycolysis after glucose injection (**Figure 2E**); whilst TNFα-treated veRA FLS showed only a modest effect, which reached statistical significance only after the addition of oligomycin, a glycolysis stimulator (**Figure 2F**).This suggests that veRA FLS may display a delay in upregulating glycolysis in response to inflammatory stimuli as previously found in other RA cell types (50). Similarly, uninflamed controls did not show a significant increase in glycolysis but displayed enhanced glycolytic capacity; suggesting that TNFα only was not sufficient to promote a glycolytic shift in these individuals. This could be explained by the fact that they were isolated from uninflamed joints with low levels of TNFα in the surrounding environment, opposite to veRA and resolving FLS that were obtained from TNF-rich inflamed joints. These findings support the hypothesis that FLS have a “metabolic memory” and display a trained immune response (51, 52) which is maintained in *ex vivo* cultures after re-exposure to inflammatory triggers, and that the FLS of resolving arthritis patients display a greater degree of metabolic plasticity in response to repeat cues. However the fact that all FLS groups upregulated glycolytic capacity following TNFα demonstrated a shared ability to enhance glycolytic flux in response to extreme stressors such as oligomycin. The extent to which repeated stimulation re-programmes metabolic pathways in FLS is of great interest and warrants further study.

Metabolic adaptation is pivotal for FLS to maintain viability and functional competence (40) and switching from glycolysis to oxidative phosphorylation is a prerequisite to promote resolution of inflammation (53, 54). In line with this, our analysis of oxidative respiration showed low steady state mitochondrial maximal and spare respiratory capacity in FLS from veRA compared with resolving arthritis. This suggests that FLS from resolving disease are better equipped to respond metabolically to inflammatory cues than those from joints which develop RA. Other groups have reported decreased oxygen consumption and maximal respiratory capacity in fibroblasts from late stage RA (21, 22). We show that this loss of mitochondrial function represents an early change in the timeline of RA disease development, suggesting that mitochondrial capacity may be linked to the ability of the joint to resolve the inflammatory state.

We also found that the resolving phenotype correlated with more dynamic mitochondrial morphology in response to TNFα, manifesting as increased connectivity (linear pixels in green) in resolving arthritis FLS compared to veRA, which displayed a more punctate/fragmented (in purple) morphology. Mitochondrial fission has an important role in inducing mitophagy in conditions of oxidative stress (55) while mitochondrial fusion is known to protect cells against deleterious mitochondrial DNA mutations (56) and enhance oxidative phosphorylation (57). Historical and recent studies have described altered mitochondrial macrostructure and high levels of mitochondrial DNA mutagenesis that correlates with inflammation in RA (19, 58). Wang et al. recently reported that synovial tissue and *ex vivo* FLS from late stage RA patients demonstrate shortened mitochondria and increased expression of the fission protein DNM1L (DRP1) (22). We did not find differences in Dnm1l mRNA levels between the groups (data not shown) suggesting that other mechanisms may drive the mitochondrial dysfunction in early disease that differ from those observed at late stage. In addition mitochondrial dynamics are regulated by activation/inflammatory stimuli (59) thus explaining the absence of mitochondrial modulation in unstimulated controls.

Correlations between mitochondrial function and age have also been demonstrated extensively, and several mechanisms have been proposed (60). Human studies have reported that mitochondrial decline advances with age and becomes particularly significant after 70 years old, with mild differences between 40 and 60 years old (61). Our veRA patients samples used for Seahorse and mitochondrial morphology analysis came from a marginally older age group (44-70, average 61) than the resolving arthritis group (32-64, average 43). Whilst we cannot definitively rule out an impact of this age difference on our measurements, we believe that the overall impact would be minor between these groups. We did not find a significant correlation between age and mitochondrial linearity (p=0.10; R=0.4) and branching (p=0.92; R=0.03) in our cohort; however a larger cohort is needed to better establish a correlation between age and mitochondrial dysfunction in RA.

The metabolic phenotypes we show to be associated with different disease outcomes were evident even after multiple passages *in vitro*, highlighting a metabolic transformation or ‘metabolic memory’ acquired by cells during the acute phase of inflammation *in vivo*. This provides a metabolic dimension to the previously described ‘imprinted aggressor’ role of the FLS (62) and offers future scope to investigate epigenetic changes and mutations in mitochondrial genetics which might confer the altered metabolism we and others have observed. Our findings suggest that the functionality of mitochondria in FLS may be of importance for the resolution of acute inflammation in the synovium. This is suggested by changes in mitochodrial morphology with increased connectivity which would indicate a tendency to rely on mitochondrial fusion or biogenesis, and oxidative phosphorylation in resolving FLS that would need further investigation. This is also in line with previous studies showing that fragmented mitochondria are the consequence of oxidative damage or are due to defects in fusion or fission (63–65). The specific mechanisms by which FLS of very early RA patients lose mitochondrial respiratory capacity and mitochondrial agility in response to TNF are yet to be determined, and this represents an intriguing challenge for future work with these difficult to obtain but important cell populations. The current study did not include patients treated with TNF blocking agents, as it focused on early phases of diseases pre-therapy. However, correlations between TNFα cytotoxicity and mitochondrial dysfunction have been reported (66–68). In RA TNFα signalling promotes increased ROS production (66) and treatment with TNF blocking agents has shown amelioration of synovial oxygen tension and reduction in mtDNA mutation as well as improvement of disease activity calculated as DAS28 CRP (69).

Taken together our findings show that FLS from resolving arthritis patients are distinct from healthy controls in terms of the consistency and magnitude of mitochondrial and glycolytic responses to an inflammatory challenge *in vitro*. In contrast, FLS from early RA patients display weaker and less consistent responses to challenge, particularly at the level of mitochondrial maximal respiration and spare respiratory capacity. These findings suggest that a lasting state of metabolic agility can be induced in FLS by exposure to inflammation *in vivo*, and that this agile state is associated with capacity to resolve. We postulate that potentiating mitochondrial function may represent a novel strategy for promoting resolution in RA and related conditions. We have recently defined multiple fibroblast subtypes in the RA joint with different functions (70). A better understanding of how the different synovial cell types co-ordinate their metabolism will be required to fully appreciate how metabolic changes in disease differs from that in health.

## Data Availability Statement

The raw data supporting the conclusions of this article will be made available by the authors, without undue reservation.

## Ethics Statement 

The studies involving human participants were reviewed and approved by Ethical approval was obtained from West Midlands - Black Country Research Ethics Committee (12/WM/0258) and all subjects provided written, informed consent. The patients/participants provided their written informed consent to participate in this study.

## Author Contributions

JF carried out metabolomic data analysis and bioenergetics experiments. JF, VP, and SC carried out data analysis and manuscript preparation. JM carried out mitochondrial imaging experiments. SR carried out cell preparations for NMR metabolomics and contributed to NMR data analysis. HA carried out cell preparations for NMR metabolomics. AP provided advice and assistance with studying cellular bioenergetics. AF and KR provided clinical samples and academic input throughout the study. SY and CB conceived and led the project. CB, SY, AF, KR, and AC provided academic support, conceptual input and manuscript editing. All authors contributed to the article and approved the submitted version.

## Funding

This work was supported by grants from the NIHR/Wellcome Trust Clinical Research Facility, University Hospitals Birmingham NHS Foundation Trust. KR, AF, and VP are supported by the National Institute for Health Research (NIHR) Birmingham Biomedical Research Centre. Support was provided by the following Arthritis Research UK Grants: Targeting fibroblasts in the treatment of inflammatory arthritis (19791), Rheumatoid Arthritis Pathogenesis Centre of Excellence grant (20298) and Arthritis Research UK Experimental Arthritis Treatment Centre (20015), Research into Inflammatory Arthritis Centre *Versus* Arthritis (grant ref 22072). AF, KR, AC, and CB are funded by the Research into Inflammatory Arthritis Centre *Versus* Arthritis (grant 22072). SC, AF, AC, and CB are funded by programme grant 21802 from *Versus* Arthritis.

## Author Disclaimer

The views expressed are those of the authors and not necessarily those of the NIHR or the Department of Health and Social Care.

## Conflict of Interest

The authors declare that the research was conducted in the absence of any commercial or financial relationships that could be construed as a potential conflict of interest.

## Publisher’s Note

All claims expressed in this article are solely those of the authors and do not necessarily represent those of their affiliated organizations, or those of the publisher, the editors and the reviewers. Any product that may be evaluated in this article, or claim that may be made by its manufacturer, is not guaranteed or endorsed by the publisher.

## Supplementary Material

Supplementary Figure 1 Pathways associated with the metabolomic profile of FLS. Pathways involved in FLS metabolism were identified using Metaboanalyst 3.0 software (71). Intensity of symbol colour and position on the y axis is representative of the pathway p value. Symbol size and position on the x axis is representative of the pathway contribution to the model. As such those pathways with high values on both axes make the greatest contribution to FLS metabolism. This model accounts for the number of metabolites represented from each pathway and not the concentration at which they are present in the samples analysed.
