# Supplementary figures and images for: Spontaneously Resolving Joint Inflammation Is Characterised by Metabolic Agility of Fibroblast-Like Synoviocytes
Source: Front Immunol. 2021 Aug 26;12:725641. doi: 10.3389/fimmu.2021.725641 (PMC8426599; doi:10.3389/fimmu.2021.725641)

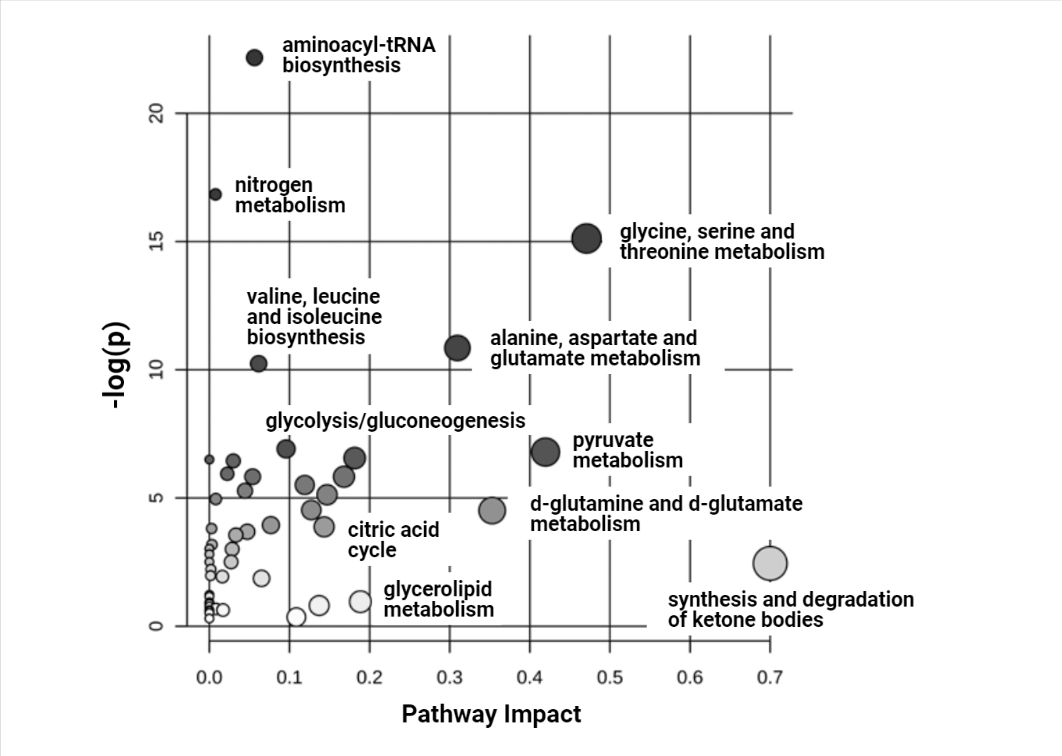

Supplement: Supplementary Figure 1 — Pathways associated with the metabolomic profile of FLS. Pathways involved in FLS metabolism were identified using Metaboanalyst 3.0 software (71). Intensity of symbol colour and position on the y axis is representative of the pathway p value. Symbol size and position on the x axis is representative of the pathway contribution to the model. As such those pathways with high values on both axes make the greatest contribution to FLS metabolism. This model accounts for the number of metabolites represented from each pathway and not the concentration at which they are present in the samples analysed. [file Image_1.jpeg]
